# Patterns and Mechanisms of Niche Partitioning Between Related Parasitoids (Hymenoptera) Sharing the Same Host Species

**DOI:** 10.3390/insects16040340

**Published:** 2025-03-25

**Authors:** Vladimir E. Gokhman

**Affiliations:** Russian Entomological Society, Moscow 111024, Russia; vegokhman@hotmail.com

**Keywords:** aggregated distribution, enemy-free space, generalist, life-history strategy, niche partitioning, oviposition, parasitic wasp, parasitoid Hymenoptera, specialist

## Abstract

Related species of parasitoid Hymenoptera often coexist on a certain host, but many details of interactions between these organisms remain unclear. The present review summarizes the main existing concepts and facts and suggests principal patterns and mechanisms that allow for the coexistence of several members of a particular parasitoid genus at the expense of the same host. Although the successful introduction of exotic parasitic wasps into the existing ecosystems often leads to the competitive displacement of related parasitoids, mere spatial and/or temporal niche partitioning between these insects is also possible. Nevertheless, many cases of coexistence of related wasp species on the same host defy simple explanations since they apparently result from complex interactions between the host and its parasitoids. The main characteristics of the oviposition process, i.e., egg volume, fecundity, and duration of the egg-laying period, are likely to correlate with other basic features of life-history strategies in parasitoid Hymenoptera. Specialist parasitic wasps often aggregate over the host patches, whereas generalists can be randomly distributed, thus reducing the degree of interspecific competition among parasitoids. However, some of the coexisting parasitic wasps, usually the weakest competitors, must also have access to enemy-free space to survive.

## Simple Summary

Related species of parasitoids often coexist on a certain host, but many details of interactions between these organisms remain unclear. The present review summarizes the main existing concepts and facts and suggests principal patterns and mechanisms that allow for the coexistence of several members of a particular parasitoid genus at the expense of the same host. Although the successful introduction of exotic parasitic wasps into the existing ecosystems often leads to the displacement of all but one related parasitoid, mere spatial and/or temporal niche segregation between these insects is also possible. Nevertheless, many cases of coexistence of related wasp species on the same host defy simple explanation, since they apparently result from complex interactions between the host and its parasitoids. The main characteristics of the oviposition process, i.e., egg volume, number of eggs, and duration of the egg-laying period, are likely to correlate with other important ecological features of parasitoids. Specialist parasitic wasps often aggregate over the host patches, whereas generalists can be distributed more evenly, thus reducing the degree of interspecific competition among parasitoids. However, some of the coexisting parasitic wasps, usually the weakest competitors, must also have access to a refuge to survive.

## 1. Introduction

One of the puzzles of parasitoid biology is how so many different parasitoid species can persist on a single host without one outcompeting the rest

D.L.J. Quicke (2015)

Parasitoid Hymenoptera is an extremely speciose, taxonomically challenging, and ecologically diverse group of insects, with the number of potentially recognized species in the world’s fauna exceeding one million [1]. Although an extensive discussion on the degree of interspecific competition between parasitic wasps took place a few decades ago [2,3,4,5,6,7,8], the crucial role of this competition in structuring host–parasitoid communities is generally accepted nowadays [9,10,11]. Moreover, Mills [12], Harvey et al. [13], and Ode et al. [14] pointed out that parasitic wasps are especially susceptible to interspecific competition due to certain features of parasitoid biology. However, despite these antagonistic interactions, sympatric species of parasitic wasps often coexist on similar or identical hosts within given habitats [15,16,17,18,19,20]. On the other hand, many published cases of the coexistence of parasitoids involve phylogenetically distant species, which often belong to different families [13,14,21,22]; however, the intensity of interspecific competition strongly and predictably increases with the degree of phylogenetic/taxonomic relatedness [23]. For example, Reitz and Trumble [24] noted that most cases of competitive displacement in parasitic wasps occur between closely related species. This apparently means that it is the competition among these parasitoids that mainly defines the result of interaction between these insects, apart from the possible influence of other organisms in a number of cases (e.g., [25,26,27]).

An initial model developed to describe the possible outcome of simultaneous host exploitation by two different parasitoids was first proposed by May and Hassell [28]. Since herbivores and other insect hosts usually exhibit aggregated distribution [29], this model demonstrated that the coexistence of the two parasitoids is possible if searching female wasps tend to aggregate in patches of higher host density. Although the probability of coexistence substantially reduces if only one of the competing wasp species contributes to stability by self-aggregation while the other searches more or less randomly, coexistence remains possible even in this case [30]. May and Hassell [28] also stated that the two parasitoids could coexist under non-equilibrium conditions, for example, when the first species outcompetes the other locally, but the second one is a better competitor at colonizing new patches. The latter species could, therefore, be labeled “fugitive” using the term earlier coined by Hutchinson [31]. Moreover, competing parasitoids can also coexist in disturbed habitats [12,32], i.e., under non-equilibrium conditions. May and Hassell [28] added that the coexistence of parasitic wasps is possible if one of them acts as an obligate or facultative hyperparasitoid of the other. Furthermore, they suggested that a refuge [5,33,34], or the so-called enemy-free space for the host (the term proposed by Jeffries and Lawton [35]), can promote stability in the host–parasitoid system [29,36,37,38], although Porter and Hawkins [39] claimed that many cases of coexistence of the specialist parasitoids could not be explained just by the host refuge effect.

The model suggested by May and Hassell [28] was explored and discussed in a number of works in subsequent years [12,30,40,41,42,43,44,45,46,47,48]. In these studies, as well as in several fundamental reviews [5,6,8,20,29] and certain empirical papers published over a much broader period of time [49,50,51,52], the main conclusions drawn by May and Hassell [28] were confirmed, and other possible conditions of the stable coexistence between multiple parasitoids attacking the same host were formulated. For instance, it was demonstrated that fluctuating temperatures (i.e., another non-equilibrium condition) might allow two parasitic wasps with different temperature optima to coexist on the same host species [49,52]. Moreover, Hackett-Jones et al. [51] reported that the coexistence of two or more parasitic wasps can also be promoted by specific differences in parasitoid phenology and other ecological traits. In addition, Comins and Hassell [30] demonstrated that persistent spatial segregation between the two coexisting parasitoids with different dispersal abilities is theoretically possible even in a completely uniform environment. In turn, Yu et al. [50] argued that parasitic wasps can coexist if they attack different stages of the same host. Nevertheless, the model developed by Briggs [44] suggested that coexistence in the latter case is possible only if a particular parasitoid can successfully attack host stages that were previously parasitized by another competing species.

Despite all the above-mentioned theoretical and empirical studies, the question of how phylogenetically close parasitoids, which exploit the same host, could coexist in sympatry via niche partitioning, remained unresolved [12,20]. This review, therefore, represents an attempt to summarize the accumulated information as well as to suggest possible answers to this question. To accomplish this task, I intentionally restrict the main scope of the present work to the analysis of interactions between closely related parasitic wasps, which belong at least to the same insect genus. On the other hand, relationships between more distant parasitoid taxa are also discussed in some cases to illustrate certain important points. Furthermore, examples considering laboratory experiments as well as observations of both indigenous and introduced parasitic wasps in the field are included to provide a broader perspective. In addition, a few important hypotheses and definitions to be used in the subsequent discussion are listed below.

## 2. Hypotheses and Definitions

### 2.1. Taxonomic Issues

Although many published host/parasitoid records inevitably contain errors in the identification of parasitic wasps and/or their hosts [53,54], the very existence of several related parasitoid species attacking the same hosts is widely accepted nowadays [20,55,56,57]. On the other hand, it seems obvious at present that a considerable number of superficially uniform parasitoid morphospecies, especially widely distributed ones, harbor so-called cryptic taxa [9,58,59,60]. These cryptic species usually demonstrate weak differences in terms of morphology but, at the same time, substantially differ in other traits, e.g., DNA sequences, cytogenetics, ecology, behavior, etc. ([61,62,63,64,65,66,67,68,69,70,71,72,73,74,75], see also [76]). Probably to a somewhat lesser extent, this often applies to host species as well [77]. All these aspects will be taken into account in the present analysis.

### 2.2. Life-History Strategies of Parasitoids

Every living species, including parasitic wasps, can be characterized by a certain life-history pattern [78]. This pattern is mainly shaped by optimal foraging and reproductive tactics, or life-history strategy, implemented by this species [79]. Various components of this strategy interact in a way that allows for the successful survival and reproduction of these organisms. However, this interaction inevitably faces more or less strong intrinsic constraints. For parasitic wasps, for instance, it is impossible to simultaneously maximize both the host-range breadth and host-use efficiency ([9,80,81], also see [82]). Another example of the constraint experienced by every biological species is the necessity to allocate resources both for sustaining adult individuals and producing progeny (see [79]). This constraint leads to an obvious trade-off, and organisms can therefore be selected for higher investments either into reproductive or somatic tissues and activities [83]. The theory of the *r*/*K* continuum, which is based on this dichotomy, eventually became perhaps the most influential and well-known biological concept among those based on the existence of the fundamental trade-off between survival and reproduction. Moreover, since *r*- and *K*-selected species evolved either towards productivity or feeding efficiency, respectively, their bionomic features include dozens of fairly predictable correlates [83]. For parasitic wasps, many correlates of that kind were listed by Force [84], who summarized the results obtained from a few detailed case studies.

Nevertheless, further research revealed substantial problems with applying this concept to the biology of parasitic wasps. Specifically, a few decades ago, Blackburn [85,86] studied relationships between various components of parasitoid life-history strategies using data from a wide array of papers on more than 470 species of parasitic wasps. He detected significant correlations between fecundity and parameters of the oviposition process, including egg volume, the rate of egg-laying, and the age of females at the beginning of oviposition [86]. Moreover, Blackburn [85] also found a positive relationship between the preadult lifespan, on the one hand, and adult size, on the other. All these traits fit the theory of the *r*/*K* continuum relatively well; however, no correlation was found between the adult lifespan and fecundity [85]. Furthermore, a positive relationship between the adult size and egg load/fecundity was revealed only when the egg size was controlled for or between closely related parasitoids [86,87]. Quite unexpectedly, an inverse correlation between the width of the so-called parasite window (i.e., the period of availability of the host for successful attack) and fecundity was also detected. All these inconsistencies and counterintuitive relationships apparently led Blackburn [86] to abandon the application of the *r*/*K* concept in its classic form to life histories of parasitic wasps in favor of the “fast-slow” continuum, referring to the high and low potential rates of population increase (also see [88]).

On the other hand, parasitic wasps are obviously not unique in demonstrating mixed lifestyle patterns. For example, combinations of ecological features that are generally considered characteristic of both *r*- and *K*-selected species were found in other patch-exploiting insects, e.g., members of the genus *Drosophila* (Diptera, Drosophilidae) [89]. Moreover, a closer study of the main parameters of the oviposition process, which appears crucial for evaluating the reproductive effort, shows that the classic theory of the *r*/*K* continuum apparently does not pay enough attention to the temporal pattern of egg-laying, apart from the egg size and fecundity. Although Pianka [83] listed semelparity and iteroparity, or, in other words, single and repeated reproduction events, as correlates of *r*- and *K*-selection, respectively, this correlation is obviously not as straightforward as it was initially supposed. Indeed, Winemiller and Rose [90] also noted substantial deviations from the classic *r*/*K* scheme when they attempted to classify the life-history strategies of North American fishes. Nevertheless, when a third axis, representing the age of maturity (i.e., the temporal aspect of oviposition), was added to the other two, reflecting fecundity and juvenile survivorship, the three main strategies, viz., periodic, opportunistic, and equilibrium, were detected. While the two latter life-history strategies are close to those observed in *r*- and *K*-selected species, the periodic one also represents a distinctive strategy. Although insects reproduce once in a lifetime, the period of egg-laying in parasitic wasps can be relatively short or extended. Furthermore, Fritz et al. [91] noted that Cole [78], who had coined the very terms of iteroparity and semelparity, tended to apply them to the patterns of egg maturation rather than egg deposition. For parasitoids, the mode of egg maturation (and hence the period of egg-laying) is characterized by the so-called “ovigeny index” (see, e.g., [92]), defined as the initial proportion of mature eggs that make up the lifetime potential fecundity. I therefore suggest that the description of life-history strategies of parasitic wasps in terms of the egg size, maximum fecundity, and duration of the oviposition period allows for a full description of the reproductive effort in these insects and, consequently, for a proper evaluation of other resources allocated for survival.

### 2.3. Generalist and Specialist Parasitoids

For the purpose of this review, the degree of parasitoid specialization is considered exclusively in terms of the host range, and a particular classification proposed by Shaw [93] is selected as a starting point. If occasional host records are excluded, this classification recognizes four categories of specialization in parasitic wasps, i.e., niche generalists, niche oligospecialists, taxon oligospecialists, and absolute specialists (see the original paper for the detailed definitions of these categories). Interestingly, these classes apparently correspond to the four sequential stages of host selection outlined by Doutt [94], namely, host habitat finding, host finding, host acceptance, and host suitability. In the present paper, however, I prefer to use two broader categories, i.e., “generalists” and “specialists” (representing niche generalists plus niche oligospecialists and taxon oligospecialists plus absolute specialists, respectively). Indeed, after an extensive debate a few decades ago [95,96,97,98,99], most experts concluded that certain parasitoids can be environmental generalists. On the other hand, highly specialized species are also detected within higher taxa of parasitic wasps ([100,101,102,103], but see [104]), although many details of host range evolution in parasitoids still remain unclear [5,20,80,105,106,107,108,109,110]. Generally speaking, related species of parasitic wasps are frequently represented by a mixture of generalists and specialists, regardless of particular evolutionary scenarios [60,111,112,113,114,115]. Although specialist parasitoids are likely to exploit their hosts more effectively than generalists (see, e.g., [116]), excessive host specialization of parasitic wasps may well drive them to extinction [117]. Nevertheless, generalist parasitoid species fairly often split into two or more specialist ones because, at a certain point, generalists apparently become unable to maintain universal life-history strategies towards the diversifying hosts [58,108].

## 3. Patterns of Niche Segregation Between Related Species of Parasitoid Hymenoptera

### 3.1. Spatial Segregation: Macroscale

A number of experts [5,12,29,48] pointed out that interspecific competition between the coexisting parasitoids must be weaker than intraspecific competition among them. However, when it comes to analyzing real patterns, especially the results of biological control programs implemented through the release of imported parasitic wasps, which are taxonomically close to indigenous ones, their outcome is generally far from straightforward. In fact, such releases of related parasitoids often result in competitive local displacement of all but one member of a particular genus [118].

For example, in North America, *Bathyplectes curculionis* (Thomson, 1887) (Ichneumonidae), which was initially introduced to control *Hypera postica* (Gyllenhal, 1813) (Coleoptera, Curculionidae), was displaced in many regions by *B. stenostigma* (Thomson, 1887) and by the much more effective *B. anura* (Thomson, 1887) [119]. The latter species apparently wins the competition due to higher fecundity, more rapid searching and handling of the host, as well as due to other bionomic features. Another European member of the family Ichneumonidae, *Pleolophus basizonus* (Gravenhorst, 1829), was introduced into the same region, where it attacks the sawfly *Neodiprion swainei* Middleton, 1931 (Hymenoptera, Diprionidae) together with the indigenous *P. indistinctus* (Provancher, 1886) [15,23]. However, *P. basizonus* apparently dominated *P. indistinctus* in most habitats, and the latter species might have already been displaced into peripheral regions [23].

Among the several imported braconid parasitoids of the subfamily Opiinae consecutively released to control *Bactrocera dorsalis* (Hendel, 1912) (Diptera, Tephritidae) in Hawaii, USA, *Fopius vandenboschi* (Fullaway, 1952) eventually replaced another larval–pupal parasitic wasp, the initially introduced *Diachasmimorpha longicaudata* (Ashmead, 1905), but, in turn, it was then displaced by an egg–pupal parasitoid that belongs to the same genus, *F. arisanus* (Sonan, 1932) [5,120]. Moreover, *F. arisanus* also generally outcompeted the native African species, *Fopius caudatus* (Szépligeti, 1913), when both parasitic wasps developed on *Ceratitis cosyra* (Walker, 1849) (Diptera, Tephritidae) in the laboratory [121]. In addition, distributions of the gregarious *Cotesia glomerata* (Linnaeus, 1758) and the solitary *C. rubecula* (Marshall, 1885) (Braconidae) in North America, which were both introduced into the USA to control *Pieris rapae* Linnaeus, 1758 (Lepidoptera, Pieridae) with an interval of about a century, were studied in much detail [122]. These parasitoids coexist in Europe because they almost exclusively use different hosts there, with *C. glomerata* usually restricted to *Pieris brassicae* Linnaeus, 1758 [123]. However, the latter species is absent in North America, where *C. rubecula* appeared to be superior in the intrinsic competition for *P. rapae* compared with *C. glomerata* [124]. This advantage can be explained, at least partly, by the action of the venom and calyx fluids of *C. rubecula*, which, either collectively or separately, inhibit egg hatching and larval development of *C. glomerata*, whereas analogous oviposition fluids from the latter species do not affect immature stages of *C. rubecula* [125]. Consequently, this parasitoid eventually displaced *C. glomerata* in many northern regions of the USA and Canada, but failed to establish in the south, probably due to disruption of the normal diapause in the former species [126].

Another classic example of species displacement among parasitic wasps can be found in the history of introductions of *Aphytis* species (Aphelinidae) aimed at controlling *Aonidiella aurantii* (Maskell, 1879) (Hemiptera, Diaspididae) in California, USA [5,61]. First, this pest was attacked by accidentally introduced *Aphytis chrysomphali* (Mercet, 1912), but the parasitoid failed to effectively suppress *A. aurantii*. However, the subsequent introduction of *A. lingnanensis* Compere, 1955 drove the previous parasitic wasp to virtual extinction in most locations. In turn, another introduced parasitoid, *A. melinus* DeBach, 1959, displaced *A. lingnanensis* in arid regions of California; nevertheless, the latter species persisted in coastal areas [127]. Generally speaking, the accumulated information shows that spatial niche partitioning between closely related parasitoids is possible on the macrogeographic scale if their populations, for instance, exist along an extended gradient of a particular abiotic factor, like temperature or humidity. Indeed, *A. melinus* and *A. chrysomphali* reportedly coexist in a number of localities in Spain. Sorribas et al. [128] suggested that this coexistence is possible due to lower spring temperatures, which allow the initially inferior but more cold-tolerant competitor, *A. chrysomphali*, to attack earlier stages of *A. aurantii*, despite their smaller size. However, Pekas et al. [129] showed that *A. chrysomphali* also changed its foraging strategy in the presence of *A. melinus* by attacking the shared host in an alternative habitat (see [130]). Similarly, five species of the genus *Ooencyrtus* (Encyrtidae), which develop inside the eggs of *Stenozygum coloratum* (Klug, 1845) (Hemiptera, Pentatomidae), coexist in various parts of the East Mediterranean [56]. A detailed study showed that the regional abundance of most of these parasitoids is apparently determined by a particular combination of average and/or extreme temperature and humidity, mediated by interspecific competition. For example, *O. telenomicida* (Vassiliev, 1904) and *O. fecundus* Ferrière et Voegelé, 1961 usually dominate in humid and semiarid regions, respectively. On the contrary, *O. pityocampae* (Mercet, 1921), which also develops inside the eggs of *Thaumetopoea pityocampa* (Denis et Schiffermüller, 1775) (Lepidoptera, Notodontidae), is found in most localities, except for the hottest/driest ones, but is relatively uncommon everywhere [56].

### 3.2. Spatial Segregation: Microscale

Other possibilities for the coexistence of parasitoids exploiting the same hosts are probably represented by more fine-grained spatial niche partitioning. For example, Ives and Hochberg [131] correctly identify spatial heterogeneity as an important potential source of enemy-free space for the hosts (also see above). Indeed, Raymond et al. [9] explicitly noted that spatial niche segregation between parasitoids that use the same host species, which results from this heterogeneity, is common in fragmented landscapes. However, I believe that the opportunities for the coexistence of two or more parasitoids depend not on environmental heterogeneity as such but rather on the varying responses of different parasitic wasps to this heterogeneity. To illustrate this view, I would like to give here two similar examples of the coexistence between egg parasitoids of Pentatomidae (Hemiptera) that belong to the families Scelionidae and Encyrtidae. Specifically, these are *Trissolcus brachymenae* Ashmead, 1887 (=*murgantiae* Ashmead, 1893) and *Ooencyrtus johnsonii* (Howard, 1898) [21] as well as *T. basalis* (Wollaston, 1858) and *O. telenomicida* [132] attacking egg clutches of *Murgantia histrionica* (Hahn, 1834) and *Nezara viridula* (Linnaeus, 1758), respectively. In both cases, *Ooencyrtus* species turned out to be superior larval competitors since the aggressive behavior of their larvae substantially decreased the fraction of host eggs parasitized by *Trissolcus* due to multiparasitism, whereas the proportion of the eggs attacked by *Ooencyrtus* was unaffected by *Trissolcus*. As a result, individuals of the latter genus were either suppressed or totally excluded from the smaller egg clutches of the host. However, members of *Trissolcus* more effectively used intact host eggs that predominated in larger patches of vegetation and/or clutches, apparently due to the higher attack rate [21,132].

It is also well known that many parasitic wasps search for their hosts within characteristic forest layers [133,134], and these preferences may differ even between closely related parasitoids. For example, Shaw [135] points out that two species of *Aleiodes* (Braconidae) of the subgenus *Chelonorhogas*, *A. pulchripes* Wesmael, 1838 and *A. rugulosus* (Nees, 1811), parasitize various members of the subfamily Acronyctinae (Lepidoptera, Noctuidae), which inhabit trees and low plants, respectively. Moreover, of the four members of *Gelis* (Ichneumonidae), which are hyperparasitoids of *Cotesia melanoscela* (Ratzeburg, 1844) in the eastern USA, three prefer to attack host cocoons at lower heights, while the fourth parasitizes cocoons higher up the trees [136]. In addition, these species tend to inhabit different trees, and when they share the same tree, they usually partition their vertical distribution. Another study [137] revealed that three *Gelis* species, i.e., *G. areator* (Panzer, 1804), *G. agilis* (Fabricius, 1775), and *G. acarorum* (Linnaeus, 1758), can attack both *C. glomerata* and *C. rubecula*. The two latter members of *Gelis* are wingless and search for hosts in grassy habitats. On the contrary, *G. areator* is fully winged and therefore forages in higher places. Moreover, all these *Gelis* species prefer to attack larger cocoons of the solitary *C. rubecula* over the gregarious *C. glomerata* [137].

A special case of niche partitioning occurs when subsequent generations of a certain host are exploited by different parasitic wasps that belong to the same genus. A few examples of this type can be found among parasitoid complexes attacking Cynipidae, in which asexual and sexual generations usually do not overlap in time and space. For instance, Forbes et al. [138] described the parasitoid guild exploiting *Belonocnema kinseyi* Weld, 1921 (=*B. treatae* auct., see [139]) in the USA. The asexual generation of *B. kinseyi* is represented by unilocular spherical galls developing on the underside of oak leaves. In contrast to this, gall wasps of the sexual generation develop on the small rootlets of these oak trees [140]. Interestingly, most parasitoids, including several species of the genera *Eurytoma*, *Sycophila* (both Eurytomidae), and *Brasema* (Eupelmidae), are exclusively restricted to the asexual generation. Although competition between these parasitic wasps, which belong to the same genera, requires further study, there are two members of the genus *Torymus* (Torymidae), *T. fullawayi* (Huber, 1927) and *T. tubicola* (Osten-Sacken, 1870), exploiting gall wasps of the sexual and asexual generation, respectively. In this case, both complete spatial and temporal niche partitioning can be observed [138]. A few decades ago, Askew [141] noted that *Torymus flavipes* (Walker, 1833) (=*auratus* auct.), a specialized parasitoid of Cynipidae on various oaks, had two generations per year, and these generations, in addition to certain differences in color, often differed in the ovipositor length. Specifically, the ovipositor was always shorter in the individuals of the second generation. Nevertheless, females of the first generation were dimorphic, and their ovipositors often were considerably longer. However, subsequent molecular analysis [109,142] demonstrated that this complex actually harbored two cryptic lineages. Substantial differences in the ovipositor length were also detected between two other members of the genus *Torymus*, *T. capillaceus* (Huber, 1927) and *T. citripes* (Huber, 1927), attacking galls of *Aciurina bigeloviae* (Cockerell, 1890) (Cynipidae) in North America [143,144].

Several examples of niche partitioning between closely related parasitoids have also been discovered among those attacking galls of another member of the family Cynipidae, *Dryocosmus kuriphilus* Yasumatsu, 1951, an invasive chestnut gall wasp (see, e.g., [145]). For instance, more than a dozen *Torymus* species, including the introduced *T. sinensis* Kamijo, 1982, were reported to exploit *D. kuriphilus* in Europe [146]. However, these parasitoids belong to various clades, which often differ in the ovipositor length [109]. Similar to *B. kinseyi*, other groups of phylogenetically close parasitic wasps that exploit *D. kuriphilus* belong to the families Eurytomidae and Eupelmidae, as well as to Eulophidae, Ormyridae, Pteromalidae, and Megastigmidae [145]. Nevertheless, their ecological relationships are predominantly unknown.

In fact, sympatric species of parasitic wasps, which exploit shared hosts and belong to the same genera but differ in the ovipositor length or specific egg placement, have been reported often in the last few decades. Perhaps the best-studied case of this kind was presented by Heatwole and Davis [147] and Gibbons [148]. These papers describe certain morphological and biological features of the three species of the genus *Megarhyssa* (Ichneumonidae), *M. atrata* (Fabricius, 1781), *M. greenei* Viereck, 1911, and *M. macrurus* (Linnaeus, 1791), which attack larvae of the horntail *Tremex columba* (Linnaeus, 1763) (Hymenoptera, Siricidae) in North America. The hosts reportedly spend their whole lives at approximately the same depth inside the logs [147]. Since the larvae apparently do not migrate, the number of available hosts remains relatively constant, providing each parasitoid species with a corresponding share of the host population. These parasitic wasps are also likely to have some form of territoriality, constantly visiting the same sites for host search, probing, oviposition, and mating (in the case of males). In addition, different parasitoid species usually develop on different logs, depending on the stage of their decay. There are also signs of temporal divergence between at least some of these parasitic wasps [147,148]. In the same region, *P. basizonus* also has a slightly longer ovipositor than *P. indistinctus* ([15], see above). Two other ichneumonids, *Reclinervellus tuberculatus* (Uchida, 1932) and *R. masumotoi* Matsumoto et Konishi, 2007, attack the same spider, *Cyclosa octotuberculata* Karsch, 1879 (Araneae, Araneidae) in Japan [149]. However, they choose different parts of the host’s abdomen for oviposition, and the adults of *R. masumotoi* emerge earlier than those of *R. tuberculatus*. This leads to earlier completion of larval development in the former species, which, therefore, usually has the upper hand if both parasitoids more or less simultaneously attack the same host [149]. Moreover, the larvae of *Phoracantha semipunctata* (Fabricius, 1775) (Coleoptera, Cerambycidae) are exploited by several members of the family Braconidae, including *Syngaster lepidus* Brullé, 1846 and three *Jarra* species [150]. Among these parasitoids, *Jarra maculipennis* Marsh and Austin, 1994 and *J. painei* Austin and Dangerfield, 1997 have shorter ovipositors and, consequently, parasitize host larvae under thinner bark than the two other species. In addition, both *Metaphycus anneckei* Guerrieri et Noyes, 2000 and *M. hageni* Daane et Caltagirone, 1999 (Encyrtidae) exploit *Saissetia oleae* (Olivier, 1791) (Hemiptera, Coccidae), but the ovipositor is significantly longer in the latter parasitoid ([151], see below).

Further analysis of the morphology and ecology of parasitoids attacking gall-inducing insects suggests other possible cases of niche partitioning among these parasitic wasps. In particular, many of them oviposit into host galls falling within the specific size range. For example, the widely polyphagous *Exeristes roborator* (Fabricius, 1793) (Ichneumonidae) is often reared from unilocular galls caused by members of the genus *Diplolepis* (Cynipidae) [152]. However, according to our observations (see [114]), it also readily attacks multilocular galls of similar size containing substantially smaller individuals of *Aulacidea hieracii* (Bouché, 1834). In addition, two European members of the genus *Eurytoma*, *E. serratulae* (Fabricius, 1798) and *E. robusta* Mayr, 1878, lay their eggs into galls of *Urophora cardui* (Linnaeus, 1758) (Diptera, Tephritidae) on thistle (*Cirsium*, Asteraceae) [153]. Endoparasitic *E. serratulae* attacks the early stages of the host, and, counterintuitively, parasitized larvae eventually induce larger galls. On the other hand, ectoparasitic *E. robusta* does not oviposit on the larvae of *U. cardui* until host chambers are formed inside the developing gall. In *E. robusta*, the tip of the female metasoma is upturned, obviously pointed, and elongated, whereas it is relatively short in *E. serratulae* [154], demonstrating that these species differ in the ovipositor length. Moreover, *E. robusta* is an important hyperparasitoid of *E. serratulae,* but the latter species does not attack *E. robusta* [153], which can also develop on *Urophora jaceana* (Hering, 1935) in the same habitat [155]. Similar relationships are observed between North American *Eurytoma gigantea* Walsh, 1870 and *E. obtusiventris* Gahan, 1934, which oviposit into the galls of *Eurosta solidaginis* (Fitch, 1855) (Diptera, Tephritidae) on goldenrod (*Solidago*, Asteraceae) [156,157,158]. Interspecific differences in the ovipositor length and the size of attacked host galls in these cases are therefore coupled with temporal niche partitioning (see the next chapter). Interestingly, closely related parasitoids often have ovipositors of different lengths if they either exploit related but non-sister host species or occupy different habitats [109,113,159].

Another complex system was described by Sivinski et al. [160], Sivinski and Aluja [161], and García-Medel et al. [162] (also see [163]). In this particular case, several members of the family Braconidae attack various flies of the genus *Anastrepha* (Diptera, Tephritidae) in Mexico. This parasitoid guild includes two members of the genus *Doryctobracon*, *D. areolatus* (Szepligeti, 1911) and *D. crawfordi* (Viereck, 1911), which differ both in the ovipositor length as well as in the range of attacked host stages. Specifically, *D. areolatus,* with the shorter ovipositor, develops inside both the middle- and last-instar host larvae, whereas *D. crawfordi* has a somewhat longer ovipositor and attacks only mature hosts. Among the four *Anastrepha* species, two, *A. fraterculus* (Wiedemann, 1830) and *A. ludens* (Loew, 1873), are exploited by both parasitic wasps. However, each parasitoid also attacks other members of the host genus, i.e., *A. alveata* Stone, 1942 and *A. bahiensis* Lima, 1937, as well as *A. obliqua* (Macquart, 1835) and *A. striata* Schiner, 1868, in the case of *D. areolatus* and *D. crawfordi*, respectively. In addition, Sivinski et al. [160] report that the former species is often considered a dominant parasitoid of *Anastrepha* throughout tropical America, whereas *D. crawfordi* is apparently limited by severe heat and low humidity but is relatively abundant at high altitudes. The same host genus is also exploited by a few species of *Diachasmimorpha*. Again, differences in the ovipositor length were found between *D. longicaudata* and *D. tryoni* (Cameron, 1911) [164].

Although this is not directly related to the subject of the present review, I believe it would also be useful to discuss here the phenomenon of the discrete variation in the ovipositor length across different generations of the same parasitoid species. At present, there are a few reports of this type. For example, the ovipositor is substantially longer in the overwintering generation of the bivoltine *Encyrtus infidus* (Rossi, 1790) (Encyrtidae), which attacks adult females of *Eulecanium caraganae* Borchsenius, 1953 (Hemiptera, Coccidae) compared to its summer generation, which oviposits into first-instar larvae of the same host [165]. In addition, an egg parasitoid of *Pyrrhalta viburni* (Paykull, 1799) (Coleoptera, Chrysomelidae), provisionally identified as *Aprostocetus* (*Chrysotetrastichus*) *suevius* (Walker, 1839) (Eulophidae), is apparently represented by two generations with ovipositors of different lengths [166,167].

### 3.3. Temporal Segregation

It seems obvious that parasitic wasps, which can attack earlier stages of the host under natural or experimental conditions, generally have a competitive advantage over other related parasitoids exploiting the same insect species [5,44]. For example, *A. melinus* can attack *A. aurantii* substantially earlier than it can be successfully exploited by *A. lingnanensis*, thus having the upper hand in the interspecific competition ([168], see above). Moreover, different time windows for oviposition often allow two or more species of parasitic wasps to coexist on the same host [10,51]. This is the case for many species of the subfamily Aphidiinae (Braconidae) [76]. Among other examples, van Baaren et al. [169,170] studied three members of the genus *Aphidius*, which attacked *Sitobion avenae* (Fabricius, 1775) (Hemiptera, Aphididae), in western Europe. These parasitoids included *A. ervi* (Haliday, 1834), *A. rhopalosiphi* De Stefani Perez, 1902, and *A. avenae* Haliday, 1834 (=*picipes* Nees, 1811). Apart from two other species, *A. rhopalosiphi* was abundant in the field all year round but usually left host patches underexploited, thus giving *A. avenae* and *A. ervi* an opportunity to coexist [170]. In addition, females of *A. avenae* never oviposited into already parasitized hosts, apart from *A. rhopalosiphi* [169]. However, relationships between *Aphidus* species, including those exploiting *S. avenae*, are apparently more complex in many cases, and they are considered below in some detail.

Carton et al. [55] described multifaceted interactions between *Leptopilina boulardi* (Barbotin, Carton et Keiner-Pillault, 1979) and *L. heterotoma* (Thomson, 1862) (Figitidae), which attacked larvae of *Drosophila simulans* Sturtevant, 1919 in Tunisia. *L. boulardi* was a better competitor, but, apart from *L. heterotoma*, it entered temperature-dependent obligate diapause, creating a time window for the latter species to exploit *D. simulans*. Additionally, *L. boulardi* and *L. heterotoma* were also reared in this location from *Drosophila melanogaster* Meigen, 1830 and *D. buzzatii* Patterson and Wheeler, 1942, respectively. Interestingly, the infection strategies of these two parasitoids also differ at the cellular level [171]. Specifically, *L. boulardi* invokes an intensive immune response of the host (while possibly suppressing it at a later stage), whereas *L. heterotoma* causes rapid lysis of the host hemocytes. Another example of different time windows for parasitoid attacks was given in the previous chapter when two *Torymus* species were found to exploit different generations of the gall wasp *B. kinseyi* [138]. In addition, Zhang et al. [172] describe relationships between two sister species of the genus *Peristenus* (Braconidae), *P. howardi* Shaw, 1999 and *P. mellipes* (Cresson, 1872), which attack true bugs of the genus *Lygus* (Hemiptera, Lygaeidae): *L. borealis* (Kelton, 1955), *L. elisus* Van Duzee, 1914, and *L. keltoni* Schwartz, 1998 in the USA. These authors found out that individuals of *P. mellipes* emerge early in June and attack the first generation of *Lygus* bugs, while *P. howardi* appears later in August and exploits the second generation of the hosts. Two other members of the same genus, *Peristenus spretus* Chen et van Achterberg, 1997 and *P. relictus* (Ruthe, 1856), which both attack *Apolygus lucorum* (Meyer-Dür, 1843) (Hemiptera, Miridae), were also studied in China. Specifically, *P. spretus* dominates over *P. relictus* in terms of the proportion of offspring emerging from the host after simultaneous oviposition of both parasitoids. However, apart from the latter species, the sex ratio in the offspring in *P. spretus* can strongly shift towards males, depending on the order of oviposition in these parasitic wasps [173].

Related parasitoids usually have similar ecological features, but substantial bionomic differences between phylogenetically close species of parasitic wasps may sometimes be observed as well. For example, both widespread parasitoids of the genus *Anisopteromalus* (Pteromalidae), *A. calandrae* (Howard, 1881) and *A. quinarius* Gokhman et Baur, 2014 [174] are ectoparasitic idiobionts, which permanently paralyze larvae of various Coleoptera, e.g., *Sitophilus granarius* (Linnaeus, 1758) (Dryophthoridae). However, host larvae attacked by *A. calandrae* usually cannot move, even if disturbed, and often die more or less quickly, whereas those exploited by *A. quinarius* remain alive for a longer time and frequently retain some degree of motility. This difference does make sense because larvae of the latter species feed on the hosts substantially longer than those of *A. calandrae* ([175], also see below).

Speaking of koinobiont vs. idiobiont parasitoids [176], the latter group is often believed to have an upper hand over koinobionts in interspecific competition because idiobionts usually interrupt host development ([13], but see [177]). Since idiobiosis on more or less concealed hosts represents an ancestral life-history strategy (see, e.g., [178]), exposed hosts apparently recruit more specialized parasitoids, and some experts believe that specialist communities of parasitic wasps are probably less structured by competition than communities of generalists ([6,7], see also [179]). However, studies of parasitoid guilds usually demonstrate that both the observed parasitoid species load of the host and parasitoid host range are substantially influenced by the sample size [5,179,180]. Nevertheless, in the case of closely related parasitoids, the competition between ecologically similar species can still be strong.

Though this is probably not temporal segregation in a strict sense, two different parasitic wasps that belong to a particular genus can sometimes be associated with the same host species, but they are constantly described as primary and secondary parasitoids. It seems obvious, however, that these parasitic wasps are not engaged in direct interspecific competition, although they are apparently involved in antagonistic ecological interactions. A good example of this situation was given by Stefanescu et al. [181], who reported *Pteromalus puparum* (Linnaeus, 1758) and *P. semotus* (Walker, 1834) (Pteromalidae) as primary and secondary parasitoids of *Vanessa cardui* (Linnaeus, 1758) (Lepidoptera, Nymphalidae), respectively.

### 3.4. Complex Cases: Disentangling Interactions Between the Components of Life-History Strategies

Although the above-mentioned case studies demonstrate relatively straightforward possibilities of niche partitioning between closely related species of parasitic wasps that attack the same host, there are also many more cases that defy simple explanations. In these cases, the coexistence of two or more parasitoids on the same host results from the complex interaction of components of their life-history strategies, usually along less obvious environmental gradients [182,183]. To illustrate this point, I will describe a few better-studied systems below.

Several decades ago, we studied the bionomics of a supposedly well-known parasitoid of the coleopteran stored-product pest *A. calandrae* (see above). However, this morphospecies turned out to harbor two reproductively isolated cosmopolitan taxa with substantial differences in external morphology and karyotype structure [184]. In addition, the ecological features of these two parasitoids, i.e., *A. calandrae* and another eventually described species, *A. quinarius* [174], were thoroughly studied in the laboratory under controlled conditions. In particular, we demonstrated that these species had alternative life-history strategies, which we interpreted in terms of the *r*/*K* continuum [175]. Furthermore, these strategies were apparently adapted to exploit their preferred coleopteran hosts with analogous ecological differences [185]. For example, *A. quinarius* can use small host patches of certain Anobiinae (Ptinidae) for a long time without exterminating them. Consequently, bionomic features of this parasitoid include shorter adult life (and hence the period of egg-laying). This shorter lifespan apparently prevents the ovipositing female from competing with her emerging daughters, although it contradicts the general characteristics of *K*-selected organisms [175]. Interestingly, there is another group of cryptic parasitoid species of the family Pteromalidae, the *Lariophagus distinguendus* (Förster, 1841) complex, which attacks the same stored-product pests [75]. However, morphological, bionomic, and genetic divergence between the members of the *L. distinguendus* complex is substantially lower than that observed between *A. calandrae* and *A. quinarius* [75,186].

Moreover, species-specific oviposition behavior of another two members of Pteromalidae, which belong to the genus *Muscidifurax* and attack pupae of *Musca domestica* Linnaeus, 1758 (Diptera, Muscidae), i.e., *M. raptor* Girault et Saunders, 1910 and *M. zaraptor* Kogan et Legner, 1970, differs similarly to *A. quinarius* and *A. calandrae* [187]. *M. raptor* has a shorter handling time and tends to parasitize the maximum proportion of individuals per patch, thus likely demonstrating certain features of *r*-selected parasitoids; in contrast, *M. zaraptor* is characterized by a longer handling time, and it usually leaves the patch after attacking just a few host individuals, therefore showing some characteristics of *K*-selected species. These different strategies apparently help the *M. raptor* and *M. zaraptor* to coexist in sympatry in North America [187]. However, competition under laboratory conditions between *M. raptor* and another introduced member of the genus, *Muscidifurax raptorellus* Kogan et Legner, 1970, often leads to a negative effect on the reproduction of the latter species, especially when hosts are in short supply [188]. Pexton and Mayhew [189] conducted laboratory experiments to study interactions between the solitary *Aphaereta genevensis* Fischer, 1966 and gregarious *A. pallipes* (Say, 1826) (Braconidae) that both attack *Drosophila virilis* Sturtevant, 1916, apparently noted similar differences between these parasitoids, which can also be described in terms of the *r*/*K* continuum. Specifically, females of *A. genevensis* almost exclusively allocate additional resources into the fat body, which results in lower fecundity and extended longevity (an apparently *K*-selected species). On the contrary, *A. pallipes* invests relatively more in reproduction and, therefore, has lower fat reserves, reduced longevity, and increased egg loads (characteristics of the presumably *r*-selected species). Nevertheless, another pattern was observed in two members of the genus *Aphidius* (Braconidae), which both exploit the grain aphid *S. avenae* ([190], also see above). Specifically, high egg production allows *A. rhopalosiphi* to attack more host individuals, while *A. avenae* invests more resources per egg laid into each aphid but does so at the expense of a lower lifespan in female parasitoids. Consequently, the life-history strategies of *A. rhopalosiphi* and *A. avenae* resemble those of *A. calandrae* and *A. quinarius,* respectively (see above). Nevertheless, all these examples collectively suggest that life-history strategies of coexisting parasitic wasps can be successfully described in terms of the oviposition process, including the duration of the egg-laying period.

The host range of a particular parasitoid is generally constant (see, e.g., [180]), but certain host species, which are shared by related parasitic wasps, are not necessarily the most important for them, especially in the case of generalists [54]. Several decades ago, Hassell and May [41] showed that a system that includes a generalist parasitoid and its host is often unstable, whereas the addition of a specialist can stabilize it. Since hosts are often distributed in patches (see above), I suggest that the specialist parasitoid, which strongly depends on these hosts, primarily aggregates within these patches. If this is the case, the coexisting generalist may be randomly distributed (see [30]). Indeed, Peters [191] found that *Nasonia vitripennis* (Walker, 1836) (Pteromalidae), which specializes in exploiting Calliphoridae (Diptera), is attracted to olfactory stimuli coming from both the host and its microhabitat, whereas the generalist *Dibrachys microgastri* (Bouché, 1834) from the same family performs rapid host search at random. In essence, this means that the searching *N. vitripennis* and *D. microgastri* females have an aggregated and random distribution, respectively, within the host habitat [191]. In turn, these behaviors demonstrated by the specialist and generalist parasitoids obviously mitigate interspecific competition between them ([114], also see [192,193]), analogous to the pattern observed among the generalist and specialist herbivores [194].

We also studied interspecific competition between two sympatric members of the genus *Eupelmus* (Eupelmidae), which attack the gall wasp *A. hieracii* [114]. This study demonstrated that those parasitoids, *E.* (*Eupelmus*) *microzonus* Förster, 1860 and *E.* (*Macroneura*) *messene* Walker, 1839, could be considered as a distributed generalist and a local specialist. These two species, therefore, represent a combination of an aggregated parasitoid and a randomly searching one that can coexist according to the models developed by May and Hassell [28] and Comins and Hassell [30] (see above). Moreover, *E. messene*, which is specialized to exploit its local host, is exclusively represented by brachypterous females [114]. This means that host-searching activities of these parasitic wasps are spatially restricted if compared to those of fully winged ones, and flightless parasitoids can exploit only those hosts that constantly have a locally high population density [136]. This concentration of host individuals is generally achieved by different mechanisms. Apart from gall wasps with multilocular galls and/or a high degree of infestation of the host plant, high host density can also be reached by the so-called morphotypical specialization [195]. In the latter case, parasitoids may attack a wide array of hosts sharing similar external appearances, apparently falling into the category of niche generalists sensu Shaw [93]. For example, wingless females of certain *Gelis* species can oviposit into small cocoons and larval cases of Lepidoptera, spider egg sacks, and cocoons of other parasitoids [20,196]. Nevertheless, morphotypical specialization is also characteristic of fully winged females of *E. microzonus*, which attack larvae of *A. hieracii* and its primary parasitoids inside the galls [114].

The morphology, biology, and DNA structure of the *Spintherus dubius* (Nees, 1834) species complex (Pteromalidae) has recently been studied in considerable detail [197]. A comprehensive molecular analysis revealed two cryptic species in this widespread complex. Moreover, the existence of the two separate taxa is also supported by a thorough morphological and bionomic study. Although both these parasitoids (provisionally designated as C1 and C2) attack weevils of the genus *Protapion* (Coleoptera, Apionidae), which, in turn, feed on several species of clover (*Trifolium*, Fabaceae), they substantially differ in terms of their specialization on particular plants. Specifically, C1 attacks hosts on several clover species, including *T. pratense* Linnaeus, whereas C2 was found only on the latter. I believe that in this case, we also have a combination of two cryptic taxa, which differ both in terms of spatial distribution and association with particular hosts and/or host foodplants. Taking all the morphological, molecular, and ecological information [197], I therefore suggest that these parasitoids, C1 and C2, could be considered as distributed generalists and local specialists, respectively, and their relationships resemble those of *E. microzonus* and *E. messene* (see above).

Although parasitic wasps attack many insects and other arthropods, they, in turn, can also become a target for hyperparasitoid species [19,198], which can substantially affect the coexistence of the primary parasitoids. Specifically, parasitic wasps may suffer a serious decline if attacked by secondary parasitoids, but, on the other hand, their presence can additionally stabilize the whole parasitoid system. In addition, hyperparasitoids can mediate the so-called apparent competition among parasitic wasps [36], which was demonstrated in the elegant experiment by van Nouhuys and Hanski [199]. When they introduced *C. glomerata* into the area where it was previously absent, the population density of the related *C. melitaearum* Wilkinson, 1937 was substantially decreased by the hyperparasitoid *G. agilis*, which can exploit both *Cotesia* species. In addition, Montovan et al. [192] hypothesized that *Hyposoter horticola* (Gravenhorst, 1829) (Ichneumonidae), which attacks larvae of *Melitaea cinxia* (Linnaeus, 1758) (Lepidoptera, Nymphalidae), underexploits host patches to escape excessive superparasitism by *Mesochorus stigmaticus* Thomson, 1886 (Ichneumonidae), thus leaving a substantial part of the host population to be attacked by *C. melitaearum*. Furthermore, high parasitoid mortality resulting from secondary parasitoids may act as a strong selection agent, which potentially increases the host specificity of these parasitic wasps (see, e.g., [9]).

At the end of this chapter, I return to the concept of the so-called fugitive species among the competing parasitoids (see above). Godfray [5] noted that alleviation of interspecific competition between these parasitic wasps is most simply achieved when the niches of competing species only partially overlap [200]. I believe that this means that at least the inferior competitor, or perhaps all parasitoid species involved in the competition, must have access to the so-called enemy-free or competitor-free space ([163,201,202,203], also see [14,122,204,205]).

### 3.5. Other Examples of Coexistence of Closely Related Parasitoids on the Same Host

Here, I collect more examples of the coexistence of related parasitic wasps that are arranged according to their taxonomic position. However, most of these cases currently await further investigation to identify the detailed mechanisms of the above-mentioned coexistence. Among koinobiont Ichneumonidae, at least three members of the genus *Diadegma*, *D. insulare* (Cresson, 1865), *D. semiclausum* (Hellén, 1949), and *D. mollipla* (Holmgren, 1868) can attack larvae of *Plutella xylostella* (Linnaeus, 1758) (Lepidoptera, Plutellidae) [206,207]. In addition, the coexistence of the two latter species in Kenya was studied in more detail. When *D. semiclausum* was introduced into this region, it eventually displaced the indigenous *D. mollipla* from most but not all locations due to the numerical dominance of its offspring under simultaneous competition with the latter species. However, *D. mollipla* is characterized by a higher proportion of the female progeny [207].

As already mentioned above, substantial numbers of related parasitoids that attack the same host are found in the braconid subfamily Microgastrinae. This apparently can be explained by their extreme species diversity and ability to control the development of the hosts. For example, the guild of parasitoids that lay eggs into larvae of *Chloridea* (=*Heliothis*) *virescens* (Fabricius, 1777) (Lepidoptera, Noctuidae) harbors both *Microplitis demolitor* Wilkinson, 1934 and *M. croceipes* (Cresson, 1872) [208]. Although these species prefer to attack the younger and older host larvae, respectively, *M. demolitor*, which usually ecloses first from the egg under equal conditions, is often outcompeted by other parasitic wasps, including *M. croceipes*. In addition, *Cotesia gonopterygis* (Marshall, 1898) and *C. risilis* (Nixon, 1974) are known as parasitoids of *Gonepteryx rhamni* (Linnaeus, 1758) (Lepidoptera, Pieridae) in Europe [57]. *C. gonopterygis* has a single generation per year and is, therefore, strictly synchronized with *G. rhamni*, whereas *C. risilis* is a multivoltine species, which apparently needs other hosts to complete its life cycle. However, many details of their coexistence are yet unknown. Similarly, the outcome of host sharing between two other polyphagous members of this genus, *Cotesia flavipes* Cameron, 1891 and *C. sesamiae* (Cameron, 1906), which attack lepidopterous borers, was studied using *Chilo partellus* (Swinhoe, 1885) (Crambidae) and *Sesamia calamistis* Hampson, 1910 (Noctuidae) [209]. It was found that *C. flavipes* outcompeted *C. sesamiae* if *C. partellus* was simultaneously parasitized by both species. Nevertheless, competition of these parasitoids on *S. calamistis* apparently resulted in the mutual survival of *C. sesamiae* and *C. flavipes*, but at a decreased rate [209]. Moreover, a separate cryptic species associated with the latter host was eventually described within the *C. sesamiae* complex [210,211,212]. Other closely related members of the genus *Cotesia*, *C. kariyai* (Watanabe, 1937) and *C. ruficrus* (Haliday, 1834) are gregarious parasitoids of several species of Noctuidae, including *Mythimna separata* Walker, 1865 [213]. Again, if females of both species oviposit into the same host more or less simultaneously, substantial numbers of larvae from mixed broods successfully develop to the final instar, then leave the host body, spin the cocoons, and pupate. Furthermore, both the solitary *Glyptapanteles porthetriae* (Muesebeck, 1928) and the gregarious *G. liparidis* (Bouché, 1834) attack larvae of *Lymantria dispar* (Linnaeus, 1758) (Lepidoptera, Erebidae) in Europe and North Africa [214]. These species do not discriminate between intact and parasitized hosts, but *G. porthetriae* can develop only as a primary parasitoid of young host larvae. On the contrary, *G. liparidis* is much more successful in attacking later host instars. However, *G. porthetriae* might have additional hosts within its distribution range to survive under these conditions.

A particular group of *Aphidius* species, which exploited the same host, *S. avenae*, was studied by Ortiz-Martínez et al. [215] in South America (also see above). This group harbored *A. ervi* and *A. rhopalosiphi*, but also included *A. uzbekistanicus* Luzhetzki, 1960. It was found that the females of *A. ervi* preferentially oviposited into the aphids already parasitized by *A. rhopalosiphi*. Ortiz-Martínez et al. [215] hypothesized that the former species thus exploited the better host detection capability exhibited by *A. rhopalosiphi*, which presumably contributed to the mutual coexistence of the three species. In addition, *A. ervi* was introduced into North America together with *A. smithi* Sharma et Subba Rao, 1959 to control *Acyrthosiphon pisum* (Harris, 1776) (Aphididae) [216,217]. Both species can recognize aphids already parasitized by any of them and generally avoid ovipositing into such hosts. However, the larva of *A. ervi* is a superior competitor, capable of killing larvae of *A. smithi*, which likely led to a substantial decline of the latter species in many regions of the USA [218].

Parasitic wasps of the family Scelionidae usually exploit aggregated hosts, e.g., egg clutches of Hemiptera and Lepidoptera. Specifically, *S. calamistis* eggs are attacked by two cryptic species, *Telenomus busseolae* Gahan, 1922 and *T. isis* Polaszek, 1993 [219]. Although both parasitic wasps could distinguish between intact and parasitized eggs, *T. busseolae* always outcompeted *T. isis* if females of both species oviposited simultaneously. Moreover, two members of the genus *Trissolcus*, *T. japonicus* (Ashmead, 1904) and *T. mitsukurii* (Ashmead, 1904), attack eggs of *Halyomorpha halys* (Stål, 1855) (Hemiptera, Pentatomidae) [220]. *T. mitsukurii* displays more aggressive behavior and spends more time defending the egg clutch if compared to the more numerous and apparently widespread *T. japonicus*. However, if *T. japonicus* coexists with *Trissolcus cultratus* (Mayr, 1879) on the same host, females of the former species generally defeat those of *T. cultratus* during fights on the egg clutches of *H. halys* [221]. Nevertheless, *T. cultratus*, apart from *T. japonicus*, can also act as a facultative hyperparasitoid of the other species.

More examples of coexistence between the related parasitoids exploiting the same host species can be found in the superfamily Chalcidoidea. For instance, several members of the genus *Gonatocerus* (Mymaridae) that attack eggs of *Homalodisca vitripennis* (Germar, 1821) (=*coagulata* Say, 1832) (Hemiptera, Cicadellidae) coexist in California, USA [222,223]. Among these parasitoids, *G. ashmeadi* Girault, 1915 is a native species, whereas *G. triguttatus* Girault, 1916 and *G. fasciatus* Girault, 1911 were imported from other regions of the country. Laboratory experiments demonstrated that these parasitic wasps differ in the age of most effectively utilized host eggs, which could allow *G. triguttatus* to coexist with *G. ashmeadi* in California. As for *G. fasciatus*, its lower parasitism rates and poorer performance indicate that it might be outcompeted by other congeneric species [222]. In addition, *G. tuberculifemur* (Ogloblin, 1957), which was also tested in the laboratory for possible use in the biological control of *H. vitripennis*, was also outperformed by *G. ashmeadi* due to its lower parasitism rate [223]. Ecological relationships of other related members of Mymaridae, *Anaphes victus* Huber, 1997 and *A. listronoti* Huber, 1997, which both attack eggs of *Listronotus oregonensis* (LeConte, 1857) (Coleoptera, Curculionidae), were studied by van Baaren et al. [224] as well as by Boivin and van Baaren [225]. Laboratory experiments showed that at least adult females of *A. victus* were able to discriminate between intact host eggs and those attacked by themselves as well as by other individuals, either conspecific or belonging to another species [224]. In addition, larvae of the solitary *A. victus* were substantially more mobile and aggressive than those of the gregarious *A. listronoti*, which, nevertheless, were able to defend themselves [225].

In many cases, parasitoids that belong to a particular genus of the family Aphelinidae attack the same host species. For example, among the members of *Encarsia*, which develop inside *Aleurocanthus woglumi* Ashby, 1915 (Hemiptera, Aleyrodidae), there is a strict primary parasitoid, *E. opulenta* (Silvestri, 1927), together with a facultative hyperparasitoid, *E. smithi* (Silvestri, 1926) [226]. To control an invasive population of *A. woglumi* in the USA, these parasitic wasps, as well as *Amitus hesperidum* Silvestri, 1927 (Platygastridae), were released in the wild. The three parasitoids consecutively progressed through the dominance of *A. hesperidum*, then of *E. smithi*, and finally of *E. opulenta*. In the end, *A. hesperidum* was totally displaced by other parasitoids, whereas *E. smithi* was found only in a restricted number of localities [226]. A few case studies involving the observed coexistence of other *Encarsia* species were cited by Viggiani [227]. Specifically, the whitefly *Aleurotuba jelinekii* (Frauenfeld, 1867) (Aleyrodidae) in Italy was attacked by several members of Aphelinidae, including *Encarsia aleurotubae* Viggiani, 1982 and *E. margaritiventris* (Mercet, 1931). However, these species were effectively displaced later when their host was suppressed by subsequently introduced *Cales noacki* Howard, 1907 (Calesidae). In addition, *Parabemisia myricae* (Kuwana, 1927) (Aleyrodidae) in Italy was mainly exploited by three parasitic wasps that belong to Aphelinidae, i.e., *Encarsia meritoria* Gahan, 1927, *E. protransvena* Viggiani, 1985 (cited as *E. transvena* auct.), and *Eretmocerus debachi* Rose et Rosen, 1992. In various regions of that country and during different periods, any of these parasitoids can become the dominant species controlling *P. myricae* [227].

Other pests of the family Aleyrodidae are also attacked by the *Encarsia* species. For example, *Bemisia tabaci* (Gennadius, 1889) in Texas, USA, was exploited by heteronomous *E. pergandiella* Howard, 1907 and thelytokous *E. formosa* Gahan, 1924, accompanied by *Eretmocerus mundus* Mercet, 1931 [18]. However, when these parasitoids attacked the host simultaneously, the strongest competition was indeed detected between the two species of *Encarsia*, which prefer to attack the same host stages ([18], also see [228]). Moreover, *E. pergandiella* prevailed over *E. formosa* in direct competition, although the latter species fed on the shared host and killed its immature stages that had already been parasitized by *E. pergandiella* [229]. Analogously, Pang et al. [230] studied the coexistence between *E. formosa* and *E. sophia* (Girault et Todd, 1915), which also exploit *B. tabaci*. It was found that the competition between these parasitic wasps apparently reduced the number of parasitoid offspring due to mutual interference. However, the overall efficiency of biological control of *B. tabaci* either remained the same or was even enhanced in some cases [230]. Furthermore, interactions of the same parasitoid, *E. formosa*, with both *E. lycopersici* De Santis, 1957 and the host species *Trialeurodes vaporariorum* (Westwood, 1856) were described by Grille et al. [231]. In contrast to *E. formosa*, *E. lycopersici* showed a higher parasitism rate on the first instar of *T. vaporariorum* than on its third and fourth instar, thus apparently having a competitive advantage over *E. formosa*. Viggiani [227] also provided a list of *Encarsia* species attacking *T. vaporariorum* in the field in Italy. This list initially included *E. tricolor* Förster, 1878, *E. inaron* (Walker, 1839) (=*partenopea* Masi, 1909) as well as *E. formosa*. However, despite all these parasitoids, *T. vaporariorum* continued to remain a considerable pest in the region. After introducing *E. pergandiella* in 1978, it soon became a leading biocontrol agent for this whitefly species. Viggiani [227] suggested it was mainly due to the ability of *E. pergandiella* to lay female and male eggs into earlier stages of the host as well as into earlier stages of other parasitoids, respectively. In addition, interactions between a conventional parasitoid *Encarsia inaron* and a heteronomous hyperparasitoid *E. tricolor*, which attacked *Aleyrodes proletella* (Linnaeus, 1758), were studied in the laboratory [232]. Specifically, both parasitoid species were introduced into the established populations of their competitors. In all cases, *E. tricolor* substantially affected the population density of *E. inaron*, which eventually decreased to very low levels. In contrast to that, *E. inaron* failed to invade the population of *E. tricolor* [232].

*Bemisia tabaci* was also used in laboratory experiments to study interactions between the two members of the genus *Eretmocerus*, *E. eremicus* Rose et Zolnerowich, 1997 and *E. mundus* [233]. Females of both species generally avoided parasitized hosts, but those of *E. mundus* did oviposit into the puparia already containing immatures of *E. eremicus*. From the latter hosts, only individuals of *E. mundus* eventually emerged [233]. In addition, several members of the genus *Coccophagus* (Aphelinidae), including *C. cowperi* Girault, 1917, *C. lycimnia* (Walker, 1839), *C. semicircularis* (Förster, 1841) and *C. rusti* Compere, 1928, attack *Coccus hesperidum* Linnaeus, 1758 and *S. oleae* (Hemiptera, Coccidae) in the USA and Europe together with a number of other parasitoids [234,235]. The precise mechanism of this coexistence is generally unknown, but heteronomous parasitism and the presence of alternative hosts, like *Coccus pseudomagnoliarum* (Kuwana, 1914), are involved, at least in some cases.

A few impressive instances of niche partitioning among phylogenetically close parasitic wasps were also found in the family Encyrtidae. Specifically, two related members of the genus *Anagyrus*, *A.* (=*Epidinocarsis*) *lopezi* (De Santis, 1964) and *A. diversicornis* (Howard, 1894), which are solitary endoparasitoids of the invasive mealybug *Phenacoccus manihoti* Matile-Ferrero, 1977 (Hemiptera, Pseudococcidae) in Africa, were imported from their native South America a few decades ago [236]. Among these parasitic wasps, *A. lopezi* successfully controlled *P. manihoti*, whereas *A. diversicornis* failed to permanently establish in the new area [237]. This is likely explained by the substantially higher search rate and percentage of female offspring in *A. lopezi* compared to *A. diversicornis*. In addition, *A. lopezi* does not demonstrate an ability to discriminate hosts attacked by *A. diversicornis*, whereas the latter species definitely possesses this feature. This apparently makes sense because *A. diversicornis* has a lower probability of survival under multiparasitism [236]. Furthermore, the coexistence of both parasitoids in South America may also be explained by the presence of an alternative host of *A. diversicornis*, *Phenacoccus herreni* Cox et Williams, 1981, which can provide a refuge for this parasitoid species [238]. Other examples of interspecific competition among Encyrtidae include several native and introduced members of the genus *Metaphycus*, which attack *C. hesperidum* and *S. oleae* ([234,235,239], also see above). In some cases, the introduction of particular species even led to the competitive displacement of native parasitoids. For example, *M. helvolus* (Compere, 1926) displaced the native *M. flavus* (Howard, 1881) on the island of Crete (Greece), whereas *M. lounsburyi* (Howard, 1898) outcompeted *M. hageni* in Israel [239]. However, certain *Metaphycus* species, like *M. angustifrons* Compere, 1957, *M. luteolus* (Timberlake, 1916), *M. stanleyi* Compere, 1940, and *M. helvolus*, as well as *M. flavus*, *M. helvolus,* and *M. lounsburyi* do coexist on these hosts in the USA and Spain, respectively. Possible mechanisms of this coexistence apparently include solitary vs. gregarious parasitism, exploitation of different host stages, presence of alternative hosts, etc.

An interesting example of niche partitioning between the two members of the genus *Torymus* (Torymidae), *T. koebelei* (Huber, 1927) and *T. baccharidis* (Huber, 1927), was discovered in western USA [2,240]. Both these species are multivoltine ectoparasitoids of *Rhopalomyia californica* Felt, 1908 (Diptera, Cecidomyiidae), but *T. baccharidis* never oviposits on the host already parasitized by *T. koebelei*. The latter species is also probably less specialized in terms of the host range. In addition, *T. koebelei* generally has higher reproductive potential, but in winter, it often goes into diapause. *T. baccharidis* and *T. koebelei* apparently also demonstrate better tolerance to lower and higher temperatures, respectively.

Females of *Trichogramma minutum* Riley, 1871 (Trichogrammatidae) spend more time on the host patch of *Ephestia kuehniella* Zeller, 1879 (Lepidoptera, Pyralidae) than those of the related coexisting species, *T. pintoi* Voegelé, 1982, but lay substantially higher proportions of male eggs in the presence of homospecific/heterospecific competitors [241].

Three members of the family Bethylidae, *Prorops nasuta* Waterston, 1923, *Cephalonomia stephanoderis* Betrem, 1961, and *C. hyalinipennis* Ashmead, 1893, attack larvae of the serious coffee pest of African origin, *Hypothenemus hampei* (Ferrari, 1867) (Coleoptera, Curculionidae) in Central America [242,243,244]. However, the most intensive contests are generally observed between congeneric females, which guard their broods. Individuals of *C. stephanoderis* are relatively more aggressive in interspecific contests, but *C. hyalinipennis* can develop as a hyperparasitoid of the two other species. Although this wasp also has a lower population growth rate, it can exploit alternative hosts and produce larger egg clutches than both *P. nasuta* and *C. stephanoderis* [243,244].

## 4. Conclusions

The parasitoid Hymenoptera is an extremely species-rich and ecologically diverse group that attacks many insects and other arthropods. It is, therefore, not surprising that related species of parasitic wasps often coexist on a certain host. Despite intensive fundamental and applied research conducted during the last decades, many details of interactions between these organisms remain unclear. The present review summarizes the main existing concepts and facts and suggests principal patterns and mechanisms, which allow for the coexistence of several members of a particular parasitoid genus at the expense of the same host. Although the successful introduction of exotic parasitic wasps into the existing ecosystems often leads to the competitive displacement of related parasitoids, mere spatial and/or temporal niche partitioning between these insects is also possible. Nevertheless, many cases of coexistence of related wasp species on the same host defy simple explanation since they apparently result from complex interactions between the host and its parasitoids. The main characteristics of the oviposition process, i.e., egg volume, fecundity, and duration of the egg-laying period, are likely to correlate with other basic features of life-history strategies in parasitoid Hymenoptera. Specialist parasitic wasps often aggregate over the host patches, whereas generalists can be randomly distributed, thus reducing the degree of interspecific competition among parasitoids. However, at least some of the coexisting parasitic wasps, usually the weakest competitors, must also have access to the enemy-free space to survive.

## Data Availability

No new data were created or analyzed in this study.
